# Effect of High-Irradiance Light-Curing on Micromechanical Properties of Resin Cements

**DOI:** 10.1155/2016/4894653

**Published:** 2016-12-04

**Authors:** Anne Peutzfeldt, Adrian Lussi, Simon Flury

**Affiliations:** Department of Preventive, Restorative, and Pediatric Dentistry, School of Dental Medicine, University of Bern, Freiburgstrasse 7, 3010 Bern, Switzerland

## Abstract

This study investigated the influence of light-curing at high irradiances on micromechanical properties of resin cements. Three dual-curing resin cements and a light-curing flowable resin composite were light-cured with an LED curing unit in Standard mode (SM), High Power mode (HPM), or Xtra Power mode (XPM). Maximum irradiances were determined using a MARC PS radiometer, and exposure duration was varied to obtain two or three levels of radiant exposure (SM: 13.2 and 27.2 J/cm^2^; HPM: 15.0 and 30.4 J/cm^2^; XPM: 9.5, 19.3, and 29.7 J/cm^2^) (*n* = 17). Vickers hardness (*H*_*V*_) and indentation modulus (*E*_IT_) were measured at 15 min and 1 week. Data were analyzed with nonparametric ANOVA, Wilcoxon-Mann-Whitney tests, and Spearman correlation analyses (*α* = 0.05). Irradiation protocol, resin-based material, and storage time and all interactions influenced *H*_*V*_ and *E*_IT_ significantly (*p* ≤ 0.0001). Statistically significant correlations between radiant exposure and *H*_*V*_ or *E*_IT_ were found, indicating that high-irradiance light-curing has no detrimental effect on the polymerization of resin-based materials (*p* ≤ 0.0021). However, one resin cement was sensitive to the combination of irradiance and exposure duration, with high-irradiance light-curing resulting in a 20% drop in micromechanical properties. The results highlight the importance of manufacturers issuing specific recommendations for the light-curing procedure of each resin cement.

## 1. Introduction

Light-curing resin-based materials currently used in dentistry polymerize as the result of irradiation with visible light in the blue or violet/blue range. The total energy of the irradiation, that is, the radiant exposure (J/cm^2^), is the product of the irradiance (mW/cm^2^) and the exposure duration (s), and numerous studies have confirmed that degree of conversion and mechanical properties of the light-cured materials increase with increasing radiant exposure [1–7]. In an effort to reduce costly chair-side time, newly marketed LED (light emitting diode) curing units offer high irradiances to allow reductions in exposure duration. Thus, most modern LED curing units operate at higher irradiances (1000–1500 mW/cm^2^) than do conventional QTH (quartz-tungsten-halogen) curing units, which might typically have irradiances of 400–500 mW/cm^2^ [8, 9]. One contemporary LED curing unit (VALO, Ultradent) features three different modes of increasing irradiance (according to manufacturer: Standard mode ≈ 1000 mW/cm^2^, High Power mode ≈ 1400 mW/cm^2^, and Xtra Power mode ≈ 3200 mW/cm^2^), the highest being much higher than that of QTH curing units and higher than that of many other LED curing units [9, 10].

One popular type of light-curing resin-based material is resin cement. These cements are primarily used for luting ceramic restorations and they most often come in dual-curing versions so as to ensure polymerization in regions where light-curing is dubious. Resin cements need to possess superior mechanical properties in order to support the overlying restoration and to avoid abrasion at the restoration margins. Among the mechanical properties, surface microhardness reflects not only the material's resistance to wear and abrasion [11] but for a given resin-based material, surface microhardness also has been shown to be an indirect measure of the degree of conversion of the polymer [12–14].

Because of the great importance of the irradiation procedure for the material properties and clinical success of the resulting restorations, it seems relevant to verify the influence of irradiance when applying irradiances that are much higher than those traditionally used. Consequently, the aim of this work was to investigate the influence of relatively high irradiances, as offered by the VALO LED curing unit, on micromechanical properties (Vickers hardness; *H*_*V*_ and indentation modulus; *E*_IT_) of resin cements. As representatives of resin cement one dual-curing, self-etch adhesive resin cement and two dual-curing, self-adhesive resin cements were chosen, and a light-curing, flowable resin composite was included as control. The null hypotheses to be tested were (1) light-curing at high irradiance and reduced exposure duration does not influence the micromechanical properties of the resin-based materials and (2) the micromechanical properties of the resin-based materials do not increase after one week of storage.

## 2. Materials and Methods

The resin-based materials used are listed in Table 1. The dual-curing, self-etch resin cement Panavia F2.0 was hand-mixed according to the manufacturer's instructions with a 1 : 1 ratio of paste A and paste B. The two dual-curing, self-adhesive resin cements (RelyX Unicem 2 Automix and SpeedCEM Plus) were used with the mixing tips delivered by the manufacturers. Panavia F2.0 and SpeedCEM Plus were stored in the refrigerator and moved to room temperature 1 h before use.

The resin-based materials were inserted into reusable Teflon molds (diameter: 10 mm; depth: 0.5 mm). The material was covered by a Mylar strip (Hawe Stopstrip Straight, KerrHawe SA, Bioggio, Switzerland) and pressed flush with the surface of the mold by means of a glass slide. The material was then irradiated through the Mylar strip at a distance of 0 mm using a custom-made device to ensure reproducible placement of the tip end of the light-curing unit. Light-curing was accomplished with a light-emitting diode (LED) VALO curing unit (Ultradent, South Jordan, UT, USA). This light-curing unit offers three modes: Standard mode (SM), High Power mode (HPM), and Xtra Power mode (XPM). The maximum irradiances as well as the spectrum of the VALO curing unit were determined for each of the three modes using a MARC PS radiometer (BlueLight Analytics Inc., Halifax, NS, Canada). The irradiances (mean values and standard deviations of ten measurements per curing mode) and the seven irradiation protocols tested are listed in Table 2 (*n* = 17 per material and protocol). Regardless of the three modes, the spectrum of the VALO curing unit ranged from 380 nm to 520 nm with a distinct smaller peak around 405 nm and a slightly stretched higher peak in the wavelength range of 450 to 470 nm.

### 2.1. Measurement of Vickers Hardness (*H*_*V*_) and Indentation Modulus (*E*_IT_)

Following light-curing and while still covered with a Mylar strip, each specimen was transferred to a black photo-resistant box to avoid any additional effect of ambient light on the polymerization process. The specimens were kept in the box at 100% humidity in an incubator at 37°C (Memmert UM 500, Schwabach, Germany) for 15 min, allowing time for the autocuring polymerization process of the dual-curing materials to proceed. The first measurement of the micromechanical properties Vickers hardness (*H*_*V*_) and indentation modulus (*E*_IT_) were then made, and *H*_*V*_ (in N/mm^2^) and *E*_IT_ (in GPa) were determined simultaneously with an automatic microhardness indenter device (Fischerscope HM2000, Helmut Fischer GmbH, Sindelfingen, Germany) in analogy to DIN 50359-1:1997-10 [15] and as previously described [16, 17]. All measurements were performed in force-controlled mode for 50 s with the test load increasing and decreasing between 0.4 and 500 mN at constant speed. The load and the penetration depth of the Vickers indenter (pyramid-shaped diamond, 136° opening angle) were measured continually during the load-unload hysteresis. *E*_IT_ was calculated from the slope of the tangent of the indentation depth curve at maximum force. WIN-HCU software (Helmut Fischer GmbH) was used for calculating the micromechanical properties. Five measurements were made on the top surface of each specimen (one central measurement surrounded by four measurements towards the periphery with a distance of ≈ 5 mm between the four peripheral indentations, each with a distance of ≈ 3 mm to the central indentation, and resulting in indentation depths of 7.5–20 *μ*m depending on material and irradiation protocol). The five measurements per specimen were averaged and thus 17 *H*_*V*_ and 17 *E*_IT_ mean values for each of the four resin-based materials and the seven irradiation protocols were available for statistical analysis. The specimens were once more transferred to black photo-resistant boxes and kept at 100% humidity in an incubator at 37°C (Memmert UM 500) for 1 week before remeasurement of *H*_*V*_ and *E*_IT_. Thus, 17 new *H*_*V*_ and *E*_IT_ mean values for each of the four resin-based materials and the seven irradiation protocols were available for statistical analysis.

### 2.2. Statistical Analysis

Because of lack of normal distribution of the data, nonparametric statistical tests were applied. *H*_*V*_ and *E*_IT_ values were analyzed with a nonparametric ANOVA according to Higgins [18] to test for significance of the three factors irradiation protocol, storage time, and resin-based material and of interactions, and the *p* values were corrected with Bonferroni-Holm adjustment for multiple testing. For* post hoc* tests, Wilcoxon-Mann-Whitney tests were performed without applying further *p* value adjustment. Spearman rank correlation coefficients were calculated between radiant exposure and single values of *H*_*V*_ or *E*_IT_ for each resin-based material and each storage time. The significance level was set at *α* = 0.05. All statistical analysis was performed with* R* 3.3.0 (The* R* Project for Statistical Computing, Vienna, Austria).

A sample size determination from preliminary tests had been performed with NCSS/PASS 2005 (NCSS, Kaysville, UT, USA) under the following conditions: effect size 2 N/mm^2^ (as the ability to detect a difference in *H*_*V*_), power of at least 80%, and level of significance *α* = 0.05 and not expecting the median outcome of one group to be higher than that of another group.

## 3. Results

**Figure 1:**
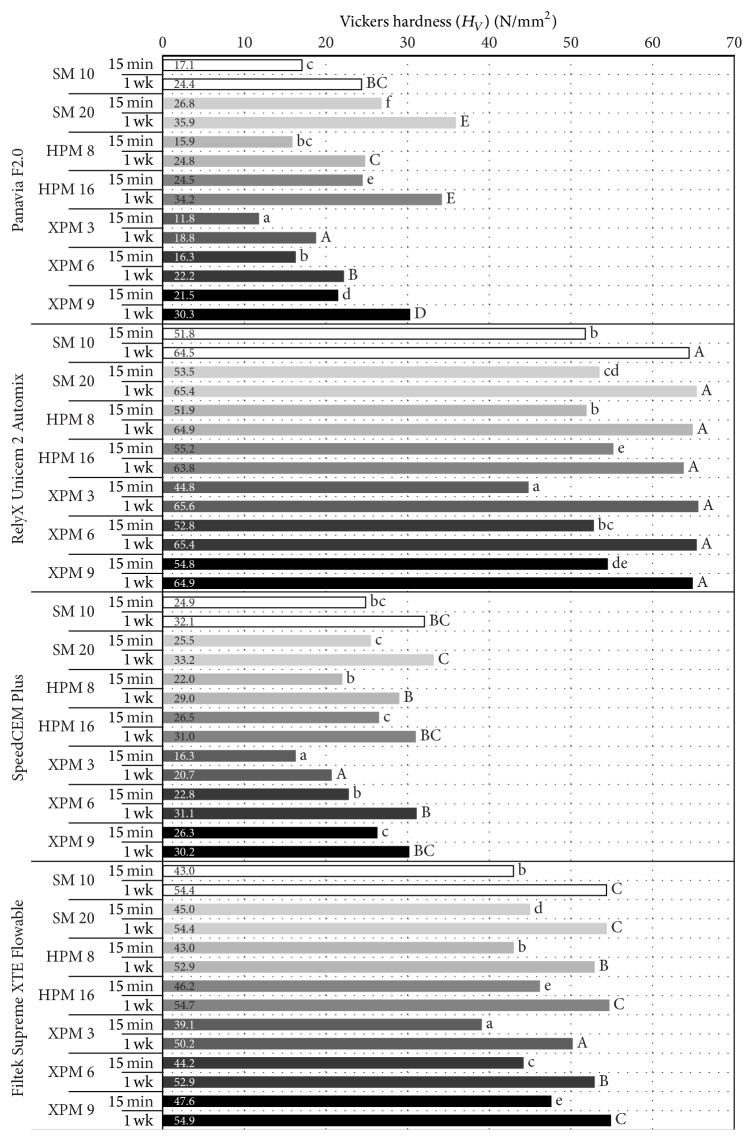
Vickers hardness (*H*_*V*_) of the four resin-based materials at 15 min and 1 week (wk) following light-curing according to one of seven irradiation protocols (SM: Standard mode; HPM: High Power mode; XPM: Xtra Power mode). Within each resin-based material, lowercase letters show the results of comparisons between the seven irradiation protocols at 15 min while uppercase letters show the results of comparisons between the seven irradiation protocols at 1 wk.

**Figure 2:**
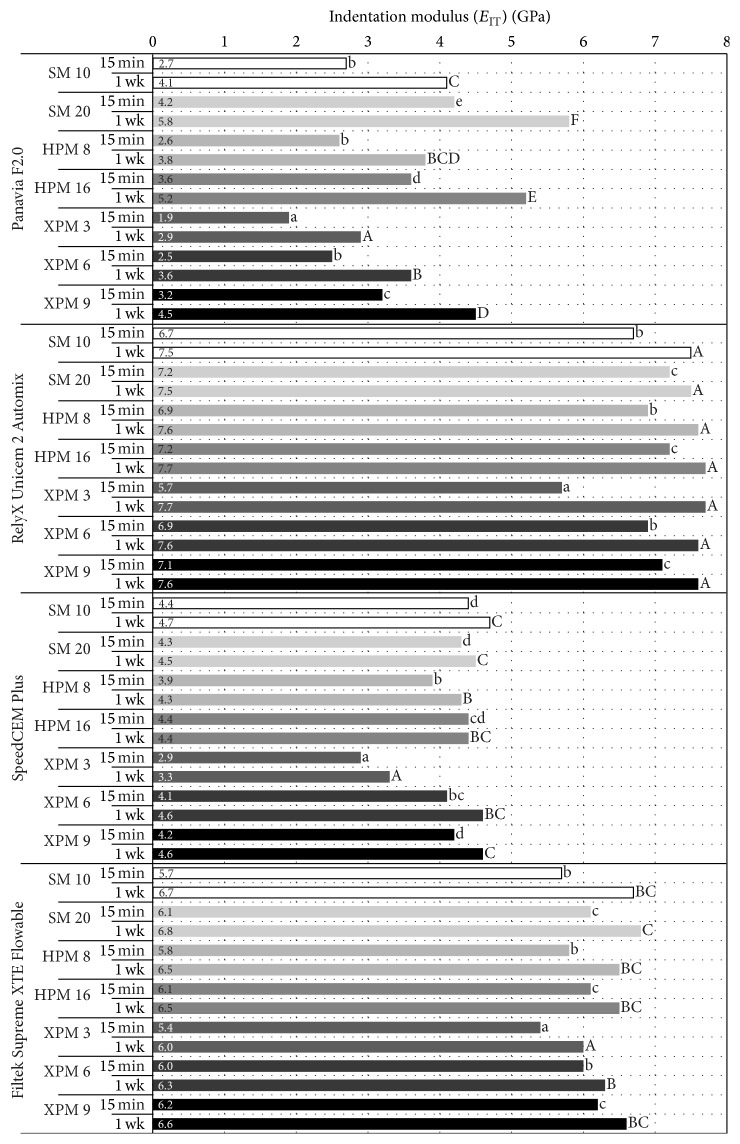
Indentation modulus (*E*_IT_) of the four resin-based materials at 15 min and 1 week (wk) following light-curing according to one of seven irradiation protocols (SM: Standard mode; HPM: High Power mode; XPM: Xtra Power mode). Within each resin-based material, lowercase letters show the results of comparisons between the seven irradiation protocols at 15 min while uppercase letters show the results of comparisons between the seven irradiation protocols at 1 wk.

The micromechanical properties determined are presented in Table 3 as median, minimum, and maximum values and in Figures 1 and 2 (medians of *H*_*V*_ and *E*_IT_). For *H*_*V*_ as well as for *E*_IT_, the ANOVA found a statistically significant effect of all three factors irradiation protocol, resin-based material, and storage time and of all interactions (*p* ≤ 0.0001).

As regards irradiation protocol, statistically significant differences in *H*_*V*_ and *E*_IT_ were found between the protocols for all resin-based materials and at both storage times with the exception of statistically similar 1 week results for RelyX Unicem 2 Automix. The results of the* post hoc* tests are displayed in Figures 1 and 2. Doubling the exposure duration (and thereby the radiant exposure) resulted in statistically significant increases (1) in *H*_*V*_ and *E*_IT_ of Panavia F2.0 for all three light-curing modes and both storage times, (2) in *H*_*V*_ and *E*_IT_ of RelyX Unicem 2 Automix and Filtek Supreme XTE Flowable Restorative for all three light-curing modes at 15 min and of Filtek Supreme XTE Flowable Restorative in HPM (*H*_*V*_) and XPM (*H*_*V*_ and *E*_IT_) at 1 week, and finally (3) in *H*_*V*_ and *E*_IT_ of SpeedCEM for HPM and XPM (*H*_*V*_ and *E*_IT_) at 15 min and for XPM (*H*_*V*_ and *E*_IT_) at 1 week. Statistically significant correlations between radiant exposure and *H*_*V*_ or *E*_IT_ were found for all resin-based materials and both storage times (*p* ≤ 0.0021) with the following three exceptions: RelyX Unicem 2 Automix at 1 week (*H*_*V*_, *p* = 0.5535; *E*_IT_, *p* = 0.5455) and Filtek Supreme XTE Flowable Restorative *E*_IT_ at 1 week (*p* = 0.1112). Indeed, the correlations were generally stronger for the 15 min results (Spearman correlation coefficients: 0.49–0.87) than for the 1 week results (Spearman correlation coefficients: 0.28–0.72).

As regards resin-based material, all four materials were found to differ significantly from one another (*H*_*V*_: *p* ≤ 0.01, *E*_IT_: *p* ≤ 0.0439) resulting in the following ranking of *H*_*V*_ and *E*_IT_ (from lowest to highest *H*_*V*_/*E*_IT_): Panavia F2.0 ≤ SpeedCEM < Filtek Supreme XTE Flowable Restorative < RelyX Unicem 2 Automix. As indicated, the ranking of Panavia F 2.0 and SpeedCEM was not clear-cut: in seven out of 28 comparisons there was no significant difference between the two materials (SM 20 15 min, *H*_*V*_: *p* = 0.4745; HPM 16 15 min, *H*_*V*_: *p* = 0.1061; HPM 8 1 week, *E*_IT_: *p* = 0.0948; XPM 3 1 week, *H*_*V*_: *p* = 0.1139, *E*_IT_: *p* = 0.1130; XPM 9 1 week, *H*_*V*_: *p* = 0.2485, *E*_IT_: *p* = 0.6098) and in four of the 28 comparisons *H*_*V*_ and *E*_IT_ of Panavia F2.0 were higher than the corresponding values of SpeedCEM Plus (SM 20 1 week, *H*_*V*_: *p* = 0.0004, *E*_IT_: *p* = 0.0001; HPM 16 1 week, *H*_*V*_: *p* = 0.0106, *E*_IT_: *p* = 0.0011).

As regards storage time, storage for 1 week resulted in significantly higher *H*_*V*_ values than those obtained after 15 min for all four resin-based materials and all seven irradiation protocols (*p* ≤ 0.0006). Likewise, 1 wk *E*_IT_ values were significantly higher than 15 min values for Panavia F2.0 (*p* < 0.0001) and Filtek Supreme XTE Flowable Restorative (*p* ≤ 0.0496) regardless of irradiation protocol, for RelyX Unicem 2 Automix (*p* ≤ 0.0005) with one exception (HPM 16, *p* = 0.0515) and for SpeedCEM Plus (*p* ≤ 0.025) with three exceptions (SM 20, *p* = 0.0537; HPM 16, *p* = 0.6542; XPM 9, *p* = 0.1791).

## 4. Discussion

The effect of irradiance protocol on the micromechanical properties of resin cements was determined using an automatic microhardness indenter device. The indentation load varied between 0.4 and 500 mN and resulted in indentation depths of 7.5–20 *μ*m that were well within the 500 *μ*m specimen height. In the clinical situation the resin cements are not exposed to air except at the restoration margins. To avoid any effect of oxygen inhibition, the resin cement specimens were therefore covered with matrices throughout the experimental period.

Within each of the three light-curing modes (SM, HPM, and XPM), doubling the radiant exposure by doubling the exposure duration generally led to significant increases in the micromechanical properties. This finding corroborates numerous previous studies on degree of conversion and mechanical properties of not only light-cured, resin-based materials [1–7], but also of dual-curing resin cements [17, 19, 20]. The fact that the dual-curing resin cements reacted similarly to purely light-curing, flowable resin composite reflects that most dual-curing resin cements rely to a large extent on effective light-curing [10, 16, 17, 21]. The finding also reflects that dual-curing resin cements seem to contain the same light-curing initiators (most often camphorquinone) as do regular light-curing resin-based materials. However, it is evident from the results of the* post hoc* analyses that the two resin cements SpeedCEM and, especially, Panavia F2.0 were more influenced by a change in the radiant exposure than were RelyX Unicem 2 Automix and Filtek Supreme XTE Ultra Flowable. Thus, doubling the radiant exposure in each of the three light-curing modes led to a mean increase in micromechanical properties of 40.1% for Panavia F2.0 while the corresponding increases for RelyX Unicem 2 Automix, SpeedCEM Plus, and Filtek Supreme XTE Flowable Restorative were 5.0%, 17.7%, and 5.3%. These results bear witness of a very efficient initiator system present in RelyX Unicem 2 Automix and Filtek Supreme XTE Ultra Flowable. The relative insensitivity of RelyX Unicem 2 is supported by studies on degree of conversion and hardness of RelyX ARC, an etch-and-rinse adhesive resin cement from the same manufacturer that also proved generally insensitive to changes in radiant exposure [22, 23]. Likewise, the relatively high sensitivity of Panavia F2.0 to changes in radiant exposure is in harmony with previous findings [17, 24]. These results may be caused by differences in the photoinitiator system indicating poorer efficiency for the latter material.

The main aim of this study was to investigate the effect of irradiation protocols using relatively high irradiances on the micromechanical properties of resin cements, that is, to answer the question “do irradiation protocols that use high irradiance for a short duration yield similar results as do irradiation protocols that use lower irradiance and correspondingly longer durations?” Due to predefined exposure durations of the light-curing unit and because rather short exposure durations were needed, which precluded individual, uncertain timing, radiant exposures were not totally identical for the three different light-curing modes. Consequently, evaluation of the effect of light-curing mode, that is, the specific combination of irradiance and exposure duration at constant radiant exposure, is not straightforward. Overall, there was a linear correlation between radiant exposure and *H*_*V*_ or *E*_IT_. Thus the “exposure reciprocity law” or “total energy principle” was generally corroborated, dictating that, at a certain radiant exposure, all combinations of irradiance and exposure duration result in comparable material properties, for example, degree of double bond conversion [2, 3, 25, 26], hardness [19, 23, 27], and modulus of elasticity [3, 28, 29]. Despite the general, significant correlation between radiant exposure and micromechanical properties and due to the significant interaction found between resin-based material and irradiation protocol, variation in the light-curing mode did not have the same impact on all four materials. A comparison between the three light-curing modes at the highest radiant exposure (SM 20 (27.2 J/cm^2^); HPM 16 (30.4 J/cm^2^); XPM 9 (29.7 J/cm^2^)) suggests that Panavia F2.0 was sensitive to the combination of irradiance and exposure duration. Indeed, high irradiance resulted in a 20% mean drop in the micromechanical properties (XPM 9 versus SM 20). Other resin-based materials have been found to react negatively to light-curing at very high irradiance and very short durations [2, 3, 30–36]. ln their study, comprising a plasma arc curing unit, Feng and Suh [31] explained the lack of reciprocity by the intrinsically higher free radical termination rate when high irradiances are applied for a short time. Later work has shown that whether or not reciprocity is upheld depends on a complex interplay between a multitude of factors such as type and concentration of initiators and viscosity and functionality of monomers as well as degree of conversion [6, 32, 35]. Consequently, the unwillingness of manufacturers to reveal the exact composition of their materials impedes proper analysis and precise explanation of the differences in behavior among the various materials. One possible explanation for the sensitivity of Panavia F2.0 to high-irradiance light-curing could be a relatively low rate and extent of polymerization [21]. In the first stages of polymerization, free radicals are terminated in a bimolecular process. As explained by Feng et al. [35] this implies that many radicals will have a shorter life time during light-curing protocols that use high irradiance and short duration and they will thus react with fewer double bonds before being annihilated. The rate of termination is reduced by immobilization of the free radicals which will prevent their mutual annihilation. At this diffusion-controlled polymerization stage, termination changes from being a bimolecular process to a monomolecular one. In case of a relatively low rate of polymerization of Panavia F2.0, the transition to monomolecular termination would be retarded and more free radicals would be terminated to result in a lower degree of conversion and in reduced micromechanical properties. As for the two least sensitive materials (RelyX Unicem 2 Automix and Filtek Supreme XTE Flowable Restorative), which are produced by the same manufacturer, they both seem to contain a very sensitive and efficient initiator system for light-curing which would give a high rate of polymerization and an earlier change to monomolecular termination. In the case of Filtek Supreme XTE Ultra Flowable, the efficiency can be explained by the content of diphenyliodonium hexafluorophosphate, a compound known to “boost” the traditional initiator system based on camphorquinone [37]. The fact that light-curing at high irradiance and reduced exposure duration impaired the micromechanical properties of Panavia F2.0 leads to partial rejection of the first hypothesis and highlights the importance of manufacturers issuing detailed recommendations concerning the light-curing procedure and of dentists accurately following these recommendations. It should also be borne in mind that, at the time of light-curing, the resin cement is covered by an indirect, ceramic, or resin composite restoration. When light passes through such restorations, its irradiance decreases due to attenuation. The decrease depends on the type, shade, and thickness of the restorative material and can be quite dramatic. Thus, using the VALO curing unit in High Power mode or Xtra Power mode, the irradiance was found to decrease by ~84% when passing through 1.5 mm discs of two types of glass ceramic and by ~95% through 3 mm discs [10]. Consequently, the negative effect of high irradiance light-curing of Panavia F2.0 may be limited to the resin cement at the restoration margins that are directly exposed to the curing light.

The four resin-based materials displayed significantly different micromechanical properties. Interestingly enough, the materials that were less sensitive to the irradiation protocol (RelyX Unicem 2 Automix and Filtek Supreme XTE Ultra Flowable) were also the materials with the highest Vickers hardness and indentation modulus. They were followed by SpeedCEM and lastly by the most sensitive material Panavia F2.0. The superior micromechanical properties of RelyX Unicem 2 Automix corroborate the findings of a previous study comprising ten dual-curing resin cements [16], and the ranking of the three dual-curing resin cements is in accordance with the ranking found by Flury et al. [17].

Following 1 week of storage, the micromechanical properties had increased for all four resin-based materials, and the second null hypothesis was therefore rejected. The polymerization process of light-cured resin-based materials continues after the end of the irradiation procedure for as long as up to 1 week, continually increasing the degree of conversion of the polymer accompanied by a strengthening of the material [38–40]. It is noteworthy that the increase in micromechanical properties from 15 min to 1 week was higher for Panavia F2.0 than for the three other materials (mean increase: Panavia F2.0, 45%; Rely Unicem 2 Automix, 19%, SpeedCEM Plus, 17%; Filtek Supreme XTE Ultra Flowable, 16%). The marked postcuring effect of Panavia F2.0 is in agreement with observations in previous studies [10, 17, 21], the latter work finding an inverse relationship between degree of conversion determined immediately following end of light-curing and the degree of conversion determined after 24 h [21].

Prior to luting of restorations with Panavia F2.0, tooth surfaces are to be treated with the accompanying self-etching ED Primer II. This primer has been shown to be essential for proper autopolymerization of Panavia F2.0 [10, 41] most likely because of its content of coinitiators [41]. Although the ED Primer II may only be incorporated in the outermost layers of the resin cement, considering the paramount importance of this primer for the polymerization of Panavia F2.0, future experiments should explore whether the ED Primer II has any influence on the sensitivity of the cement to high-irradiance light-curing.

## 5. Conclusions

This study has shown that the irradiation protocol followed when light-curing dual-curing resin cements significantly influences their micromechanical properties. Overall, there was a linear correlation between radiant exposure and *H*_*V*_ or *E*_IT_, indicating that high-irradiance light-curing has no detrimental effect on the polymerization of resin-based materials. However, one resin cement was negatively affected by light-curing protocols using very short exposure durations at very high irradiances, highlighting the importance of specific recommendations for the light-curing procedure of each product.

## Figures and Tables

**Table 1:** Resin-based materials used (manufacturers' information).

*Panavia F2.0* (TC) Kuraray Noritake Dental Inc., Okayama, Japan	LOT-Nr: AU0119 (Paste A)/BG0054 (Paste B) Paste/Paste	

Type of resin-based material	Dual-curing (self-etch) adhesive resin cement	

	*Paste A*	*Paste B*

Methacrylates	% volume n.a./% weight n.a.	% volume n.a./% weight n.a.

Type of methacrylates	(i) 10-Methacryloyloxydecyl dihydrogen phosphate (MDP) (ii) Hydrophobic aromatic dimethacrylate (iii) Hydrophobic & hydrophilic aliphatic dimethacrylate	(i) Hydrophobic aromatic dimethacrylate (ii) Hydrophobic & hydrophilic aliphatic dimethacrylate

Initiators, stabilizers, and pigments Type of initiators	% volume n.a./% weight n.a. (i) dl-Camphorquinone	% volume n.a./% weight n.a. n.a.

Filler	(i) Silanated silica (ii) Silanated colloidal silica	(i) Silanated barium glass (ii) Surface treated sodium fluoride

Filler particle size	(i) Total filler content: 59% volume	
	0.04–19 *μ*m	

*RelyX Unicem 2 Automix* (A3O)	LOT-Nr: 610318	
3M ESPE, Neuss, Germany	Paste/Paste (Automix)	

Type of resin-based material	Dual-curing, self-adhesive resin cement	

	*Base*	*Catalyst*

Methacrylates	% volume n.a./% weight n.a.	% volume n.a./% weight n.a.

Type of methacrylates	(i) Triethylene glycol dimethacrylate (TEGDMA) (ii) Methacrylate monomers (iii) Methacrylates with phosphoric acid groups	(i) Methacrylate monomers

Initiators, stabilizers, and pigments	% volume n.a./% weight n.a.	
Type of initiators	(i) Camphorquinone	

Filler	(i) Glass powder (ii) Silane treated silica	(i) Silane treated glass (ii) Silane treated silica

Filler particle size	(i) Total filler content: 43% volume	
	90%*～*12.5 *μ*m	

*SpeedCEM Plus* (Yellow)	LOT-Nr: U51719	
Ivoclar Vivadent AG, Schaan, Liechtenstein	Paste/Paste (Automix)	

Type of resin-based material	Self-adhesive, self-curing resin cement with light-curing option	

	*Base*	*Catalyst*

Methacrylates	% volume n.a./% weight n.a.	% volume n.a./% weight n.a.

Type of methacrylates	(i) Urethane dimethacrylate (UDMA) (ii) TEGDMA (iii) Polyethylene glycol dimethacrylate (PEG-DMA) (iv) Methacrylated phosphoric acid ester (v) 1, 10-Decandiol dimethacrylate, copolymers	

Initiators, stabilizers, and pigments Type of initiators	n.a. n.a.	n.a. Dibenzoyl peroxide

Filler	% volume n.a./% weight n.a. (i) Ytterbium trifluoride (ii) Barium glass (iii) Silicon dioxide	

Filler particle size	(i) Total filler content: ~45% volume	
	0.1–7 *μ*m (mean: 5 *μ*m)	

*Filtek Supreme XTE Flowable Restorative* (A3) 3M ESPE, St. Paul, MN, USA	LOT-Nr: N761493 Paste	

Type of resin-based material	Light-curing, flowable resin composite	

Methacrylates	% volume n.a./% weight n.a.	

Type of methacrylates	(i) Bisphenol A-glycidyl methacrylate (Bis-GMA) (ii) TEGDMA (iii) Procrylat resins	

Initiators, stabilizers, and pigments	% volume n.a./% weight n.a	
Type of initiators	(i) Benzotriazol (ii) Ethyl-4-dimethyl aminobenzoate (iii) Camphorquinone (iv) Diphenyliodinium hexafluorophosphate	

Filler	(i) Total filler content: ~46% volume/~65% weight	

Filler particle size	(i) Ytterbium trifluoride (0.1–5 *μ*m) (ii) Nonagglomerated/-aggregated surface modified 20 & 75 nm silica (iii) Modified aggregated zirconia (4–11 nm)/silica (20 nm) cluster (average cluster particle size: 0.6–10 *μ*m)	

n.a. = not applicable (no further/detailed information of manufacturer available).

**Table 2:** Irradiances (mean values (standard deviations), *n* = 10), exposure durations, and resulting radiant exposures of the seven irradiation protocols included.

Irradiation protocol	Irradiance (mW/cm^2^)	Exposure duration (s)	Radiant exposure (J/cm^2^)
SM 10	1324 (45.3)	10	13.2
SM 20	1361 (35.8)	20	27.2
HPM 8	1871 (52.2)	8	15.0
HPM 16	1899 (42.4)	16	30.4
XPM 3	3162 (88.2)	3	9.5
XPM 6	3213 (110.9)	6	19.3
XPM 9	3299 (89.0)	9	29.7

**Table 3:** Vickers hardness (*H*_*V*_) and indentation modulus (*E*_IT_) (minimum, median, and maximum values; *n* = 17) at 15 min and 1 week (wk) following light-curing according to one of seven irradiation protocols.

			Resin-based materials															
			Panavia F2.0				RelyX Unicem 2 Automix				SpeedCEM Plus				Filtek Supreme XTE Flowable Restorative			
VALO modes	Irradiation protocol	Values	*H*_*V*_ (N/mm^2^)		*E*_IT_ (GPa)		*H*_*V*_ (N/mm^2^)		*E*_IT_ (GPa)		*H*_*V*_ (N/mm^2^)		*E*_IT_ (GPa)		*H*_*V*_ (N/mm^2^)		*E*_IT_ (GPa)	
			15 min	1 wk	15 min	1 wk	15 min	1 wk	15 min	1 wk	15 min	1 wk	15 min	1 wk	15 min	1 wk	15 min	1 wk
Standard mode (SM) (one activation = 5 s)	SM 10	Maximum	21.9	30.8	3.5	5.5	54.0	70.2	7.2	8.3	29.9	38.3	5.1	5.8	43.9	57.8	6.2	7.3
		**Median**	**17.1**	**24.4**	**2.7**	**4.1**	**51.8**	**64.5**	**6.7**	**7.5**	**24.9**	**32.1**	**4.4**	**4.7**	**43.0**	**54.4**	**5.7**	**6.7**
		Minimum	14.7	20.2	2.2	2.7	49.3	61.5	5.5	6.6	18.7	24.1	2.9	3.7	41.4	51.8	5.3	6.2
	SM 20	Maximum	29.9	41.5	4.7	6.3	55.8	71.4	7.7	8.6	31.1	36.2	5.3	5.6	47.0	60.8	6.4	7.2
		**Median**	**26.8**	**35.9**	**4.2**	**5.8**	**53.5**	**65.4**	**7.2**	**7.5**	**25.5**	**33.2**	**4.3**	**4.5**	**45.0**	**54.4**	**6.1**	**6.8**
		Minimum	21.3	29.8	3.4	4.5	50.3	60.5	6.5	7.0	22.8	26.8	3.7	4.1	43.7	52.3	5.5	6.0

High Power mode (HPM) (one activation = 4 s)	HPM 8	Maximum	22.5	30.6	3.6	5.2	54.0	70.4	7.2	8.2	28.6	34.5	4.9	5.2	45.4	57.6	6.2	7.4
		**Median**	**15.9**	**24.8**	**2.6**	**3.8**	**51.9**	**64.9**	**6.9**	**7.6**	**22.0**	**29.0**	**3.9**	**4.3**	**43.0**	**52.9**	**5.8**	**6.5**
		Minimum	14.7	20.5	2.3	2.9	49.8	61.0	4.9	6.7	18.8	25.1	3.3	3.8	41.1	46.9	5.3	5.5
	HPM 16	Maximum	27.6	42.7	4.4	6.3	59.0	67.3	7.6	8.0	31.4	36.5	5.5	5.3	48.5	60.9	6.5	7.2
		**Median**	**24.5**	**34.2**	**3.6**	**5.2**	**55.2**	**63.8**	**7.2**	**7.7**	**26.5**	**31.0**	**4.4**	**4.4**	**46.2**	**54.7**	**6.1**	**6.5**
		Minimum	21.8	27.8	3.0	4.0	52.7	55.5	6.6	6.8	20.4	27.7	2.8	4.0	44.3	52.7	5.4	5.4

Xtra Power mode (XPM) (one activation = 3 s)	XPM 3	Maximum	15.1	25.0	2.4	4.7	47.0	68.8	6.3	8.1	20.8	33.5	3.9	5.0	40.8	55.3	5.6	6.9
		**Median**	**11.8**	**18.8**	**1.9**	**2.9**	**44.8**	**65.6**	**5.7**	**7.7**	**16.3**	**20.7**	**2.9**	**3.3**	**39.1**	**50.2**	**5.4**	**6.0**
		Minimum	9.1	15.2	1.4	2.3	42.8	60.8	5.2	6.6	10.1	13.0	1.9	2.1	36.5	45.2	4.6	5.3
	XPM 6	Maximum	18.4	29.4	2.9	4.5	56.8	72.7	7.3	8.6	26.1	33.5	4.6	5.2	46.6	57.0	6.2	6.9
		**Median**	**16.3**	**22.2**	**2.5**	**3.6**	**52.8**	**65.4**	**6.9**	**7.6**	**22.8**	**31.1**	**4.1**	**4.6**	**44.2**	**52.9**	**6.0**	**6.3**
		Minimum	13.5	17.0	2.3	2.5	48.5	59.6	6.0	7.0	18.3	24.3	3.4	3.7	42.5	50.2	5.3	5.6
	XPM 9	Maximum	25.1	37.5	3.8	6.0	59.8	75.6	7.5	8.1	33.2	36.5	5.7	5.2	49.8	57.5	6.7	7.4
		**Median**	**21.5**	**30.3**	**3.2**	**4.5**	**54.8**	**64.9**	**7.1**	**7.6**	**26.3**	**30.2**	**4.2**	**4.6**	**47.6**	**54.9**	**6.2**	**6.6**
		Minimum	19.8	21.8	2.8	2.9	52.5	61.1	6.2	7.0	20.8	27.6	3.6	3.8	45.3	51.7	5.4	5.3
